# Conducting Physical Activity Research on Racially and Ethnically Diverse Adolescents Using Social Network Analysis: Case Studies for Practical Use

**DOI:** 10.3390/ijerph191811545

**Published:** 2022-09-14

**Authors:** Tyler Prochnow, Meg Patterson, M. Renée Umstattd Meyer, Joseph Lightner, Luis Gomez, Joseph Sharkey

**Affiliations:** 1 School of Public Health, Texas A&M University, College Station, TX 77843, USA; 2 Robbins College of Health and Human Sciences, Baylor University, Waco, TX 76798, USA; 3 School of Nursing and Health Studies, University of Missouri-Kansas City, Kansas City, MO 64108, USA

**Keywords:** social influence, quantitative methods, data collection, adolescent, friendship networks, family systems, systems science

## Abstract

Adolescent physical activity (PA) is significantly impacted by peer behaviors through peer influence, peer selection, and popularity. However, the scales for these social constructs may not fully capture the detailed social networks and mechanisms responsible for PA behavior changes. This level of detail and granularity can be quantified and analyzed through social network analysis (SNA). To demonstrate the variety, utility, and efficacy of SNA in adolescent PA research, this article aims to provide four case studies on the collection of social network and PA data on ethnically and racially diverse adolescents. Through case studies, this article provides tangible ways in which SNA can be used to evaluate social influences on PA behaviors. Case studies are presented on: (1) Youth Engagement in Sport—an egocentric analysis of middle school youth participation in an experiential sport program with 3- and 6-month follow-ups; (2) Summer care program networks—an egocentric and whole network longitudinal study of adolescents at summer care programs; (3) The Convoy method—a qualitative egocentric discussion activity with adolescents from *colonias* on the Texas-Mexico border; and (4) A father-focused, family-centered health program—an egocentric experimental analysis of children participating in a health program. Data collection procedures are listed and example surveys are provided. Descriptive analyses are included, as are recommendations on further analysis techniques for each type of network data. Using SNA, researchers can understand social contexts in a more specific manner, better positioning interventions to alter such influences.

## 1. Introduction

Despite the numerous health benefits stemming from regular physical activity (PA) [1], only 26.1% of adolescents in the United States reported they had engaged in PA for 60 min on each of the last 7 days [2]. Adolescents under 18 years of age are recommended to be physically active for at least 60 min each day at a moderate to vigorous intensity level and participate in muscle-strengthening activity on three or more days each week [3,4]. Disparities in PA attainment have been noted between racial and ethnic groups, as adolescents of color (specifically Black and Latino adolescents) are significantly less likely to meet PA recommendations compared to non-Latino White adolescents [5,6,7]. 

Adolescent PA is significantly impacted by peer behaviors, namely, through peer influence, peer selection, and popularity [8,9,10]. Adolescents may become friends with others who are similar to themselves in terms of their PA behavior. As a result, they may become more similar to their friends regarding both the type of PA and amount of time spent being physically active [8,9]. Popularity can also be a driving force behind adolescent health behavior, as being more popular may predict the ability to influence or be influenced by social norms [10]. Therefore, there is a need to comprehensively understand how social connections may impact health behavior, specifically, adolescent PA. 

Health behavior scholars have expressed a need to look beyond individual characteristics to better understand the impact of social connections and social structure on health behavior [11,12,13]. The theoretical basis to understand these connections in health behavior is supported by several theories, including reciprocal determination in social cognitive theory [14], family influences in family systems theory [15], and interpersonal influences in social ecological models [16,17,18]. Often, social environments are measured using scales or constructs of social connectedness, social support, social capital, or social norms [19,20]. However, scales for these social constructs may not fully capture the detailed social networks and influence mechanisms which are responsible for PA behavior change. This level of detail and granularity can be quantified and analyzed through social network analysis (SNA) [21]. 

**Figure 1:**
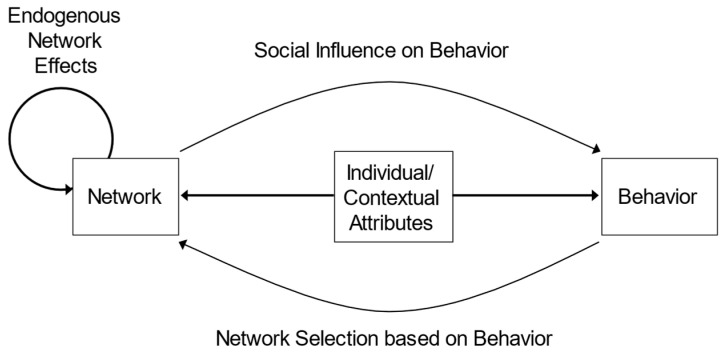
Framework for how networks may impact health behavior.

SNA allows researchers to investigate the influences of the social environment on a more granular level by understanding specific connections between individuals and how those connections are associated with, and at times, drive, behavior [21]. SNA is a set of theories and methods to analyze and understand the social influences and structures that affect an individual and their health behaviors [22]. The analytical focus is placed on the connections between individuals and what that may mean for health [21]. From these connections, researchers can understand how behavior is spread, how social structures impact health, and how individuals influence and befriend others [21]. Figure 1 provides a visual representation of how networks may impact health behavior.

Researchers have consistently found a connection between adolescent PA and their social networks [8]. However, few researchers have obtained formal training regarding the use of a suitable methodology to analyze such observations [23]. SNA requires additional expertise in data collection procedures and data analysis, which may inhibit researchers from applying it [24]. As interest in SNA to better understand adolescent PA is growing, resources illuminating the collection of SNA data in adolescent PA research are needed. Specifically, this article fills this gap in understanding and is designed to be a resource for PA researchers who are interested in using SNA in their own work. This manuscript describes tangible ways in which SNA has been used to evaluate PA in adolescents.

### 1.1. SNA Overview

SNA conventionally consists of two analytical approaches: egocentric and whole network research [21]. The egocentric approach centers on the perception of the individual being surveyed (also termed the “ego”). Hence, egocentric approaches consider the social environment of the individual, i.e., the closest and most personal connections (i.e., “alters”). To understand these connections, respondents are asked to report a set of alters that are meaningful to them (e.g., people with whom they feel close; people they go to for advice), and provide characteristics about each alter (e.g., relation to ego, gender) [24]. The individual will then be asked to report on their own behaviors and their perception of the behaviors of their nominated alters. From these questions, network concepts such as composition, homophily, and density can be derived (see Table 1 for a summary of network concepts and definitions). On the other hand, in whole network analyses, each member of the network is asked to report their own behaviors and only connections to the other individuals within the network [21]. Whole network research is an approach that accounts for an entire, defined network, i.e., all connections between individuals within a given boundary (e.g., classroom, summer care program [21]). Network variables drawn from whole network research include centrality (e.g., degree centrality, betweenness centrality), group-based measures (e.g., k-cores), and network structure (e.g., density). Table 1 summarizes all network variables and definitions. 

In both approaches, data collection and analysis are focused on dyadic structures (i.e., the connection between two people). To generate sets of dyads that together form networks, network generator questions are used [24]. For example, researchers conducting whole network research might provide a roster of all members of a network to each respondent and ask them to indicate anyone in their defined network they play with at school. Similarly, in egocentric network research, an investigator might ask respondents to indicate anyone with whom they have actively played in the last week, eliciting a set of dyads which is meaningful to each respondent [25]. Further, in egocentric approaches, it is also necessary to ask name interpreter questions which allow the researcher to collect information on the alters of each ego. For example, after developing a list of alters the adolescent plays with, a researcher could ask specific questions to find out more about each person, such their gender, what their relationship to the ego is, and how often the ego plays with that alter. 

### 1.2. Purpose

This article aims to provide four case studies on the collection of social network and PA data regarding ethnically and racially diverse adolescents. Through these case studies, this article will provide tangible ways SNA can be used to evaluate social influences on PA behaviors. By understanding how SNA data can be collected in relation to PA, this article aims to encourage more refined use of this research technique in PA research including future intervention programming using network approaches.

## 2. Materials and Methods

Below, we demonstrate four examples of how to conduct PA research employing one or both of the SNA approaches described above. For each case study, we describe the background and sample, PA assessment, SNA approach, and descriptive network and PA results. Specifically, egocentric network composition scores are generated to describe the networks in all studies. Means and standard deviations for all PA measures are also calculated. SPSS v.28 was used for data cleaning and analysis [26]. Table 2 provides study design and sample characteristics for each of the case studies. All studies received Institutional Review Board approval prior to data collection and utilized both adolescent ascent and adult consent procedures. 

### 2.1. Case Study 1: Youth Engagement in Sport

#### 2.1.1. Study Description and Sample

This study aimed to understand the social network factors associated with PA among middle school youth in an urban setting as part of the baseline assessment for an experiential PA promotion program. Students from three inner city, low-income, minority serving middle schools (aged 10–13) in Kansas City, Missouri, were recruited to participate in an after-school, sports sampling intervention with the main goal of increasing PA [27]. Youths were recruited by school staff, through flyers and at school events (e.g., enrollment, parent-teacher conferences, and other events). The youths filled out electronic surveys at baseline, at 3-months, and at 6-months. A detailed description of the intervention can be found elsewhere [27].

#### 2.1.2. PA Assessment

PA was assessed in this case study via self-report. Participants self-reported leisure time PA for vigorous-intensity, moderate-intensity PA, walking, and sedentary behavior via the International PA Questionnaire short form [28,29]. The IPAQ-SF was collected via a researcher-administered, in-person tablet. Time spent engaging in moderate- to vigorous-intensity PA (MVPA) was calculated via scale recommendations.

#### 2.1.3. SNA Approach

Using an egocentric approach, youths were asked to report up to five of their closest friends based on free recall. For each friend, participants were asked how many hours per week they thought each friend was physically active and how many hours per week they engaged in physical activity in-person and virtually with each friend. The participants could respond “none”, “about half an hour per week”, “about one hour per week”, “about 2–3 h per week”, “about 4–6 h per week”, and “about 7 h per week”. Youths were also asked if each friend encouraged them to be physically active (e.g., suggest going to the park, playing basketball or soccer); they could respond “never”, “sometimes”, or “always”. Lastly, youths were asked how close they feel to each of their friends; they responded on a 5-point Likert scale from “very close” to “not at all close”. Table S1 presents the social network questions from this study.

### 2.2. Case Study 2: Summer Care Program Social Networks

#### 2.2.1. Study Description and Sample

This study was designed to understand the social dynamics present among adolescents at summer care programs with regard to PA. Adolescents aged 8–12 years old from two summer care programs (i.e., Boys & Girls Clubs) in Waco, Texas and Kenosha, Wisconsin were invited to participate in researcher-administered surveys at the start (time 1) and end (time 2) of summer (8 weeks between time points). A research assistant used a computer (using EgoWeb2.0) to facilitate the survey at the program during normal program hours in a room away from the main activities. Each site was analyzed as its own separate whole network. Adolescents self-reported age, sex, race, and ethnicity prior to being asked about PA or network data.

#### 2.2.2. PA Assessment

PA was assessed by using a self-reported measure from the World Health Organization’s Health Behavior School Aged Adolescents survey [30]. Adolescents self-reported the number of hours they were physically active in the last week with possible response options: “none”, “about half an hour per week”, “about one hour per week”, “about 2–3 h per week”, “about 4–6 h per week”, or “about 7 h per week or more”, coded respectively on a scale of 0–5. Test-retest reliability has been shown to be acceptable with samples of adolescents [30].

#### 2.2.3. SNA Approach

This study used a hybrid (whole network and egocentric together) SNA approach. Based on a network-generator question used in a study investigating adolescent PA and after-school social networks [31], participants were asked to report the names of up to five peers with whom they socialized, talked to, and/or did things with the most at the summer care program (whole network). Adolescents were provided a full roster (in the form of a list of names) of all 8–12 year-olds at the summer care program. If an adolescent was enrolled in the program anytime during the summer, they were added to the list for the second time point. Adolescents were also asked to report the names of up to five people outside the program (egocentric network) with whom they interacted the most. Participants provided information about each alter they had nominated, including their sex (“boy” or “girl”), age, relationship (“sibling”, “friend”, “relative”), where they live (“in household”, “in neighborhood”, “outside neighborhood”, or “don’t know”), how often they actively played with the alter (“often”, “sometimes”, “rarely”), how many hours they thought the alter was active each week (“none”, “about half an hour per week”, “about one hour per week”, “about 2–3 h per week”, “about 4–6 h per week”, or “about 7 h per week or more”), and if the alter helps them to be active (“yes”, “no”). Although these data were collected on a computer, a paper version of this tool is presented in Table S2.

### 2.3. Case Study 3: Convoy Method among Colonia Youth: Qualitative

#### 2.3.1. Study Description and Sample

Individuals aged 7–11 years old from *colonias* on the border of Texas and Mexico were invited to participate in this study. *Colonias* are defined as economically distressed communities consisting of persistently low or very-low income households based on the Federal poverty index, located at or near the U.S.-Mexico border area with an outer range stretching from 50–150 miles into the U.S. [32]. According to the U.S. Department of Health and Human Services, the U.S.-Mexico border region is medically underserved, with increased social and health barriers, increased rates of poverty, and disproportionate rates of disease [33,34]. Through a parental consent and adolescent assent process, each adolescent was asked to complete one short survey (administered by a *promotora*-researcher) and participate in three group discussions with other adolescents of his/her age, which included activities like drawing, mapping, and photography. The purpose of the study was to learn more about everyday food and activity places in their communities. The short survey took 15-min to complete, while each discussion group lasted approximately 2 h. Adolescents were eligible to participate for this study by being of Mexican heritage (grandparent, parent, and/or adolescent born in Mexico), 7–11 years of age, prefer to read, write, and speak in Spanish, resided in his/her *colonia*/neighborhood for at least two years, and living in a household with at least one parent or caregiver. Each group discussion was audio recorded and two-stage transcribed and translated by the Support & Data Management Linguistics Team. 

The administered adolescent survey collected basic demographic information about each adolescent (date of birth, gender, current grade level, race/ethnicity, country of birth, and household composition), as well as language preferences (to speak/read, with parents, at home), participation in summer meal programs, nutrition and food safety, and PA behaviors (exercise, sports, active living). As for the three group discussions, the first focused on nutrition and dietary behaviors (thoughts on food, meals, and snacks at home, school, neighborhood, or community), the second on PA (exercise, active play, walking, and sports), and the third used a photo elicitation process to discuss both nutrition and PA behaviors. Although we just described the overall structure of this study, in this publication, we strictly focus on the second group discussion (PA alone), its components, and process. Twelve separate discussions were analyzed for this study involving a total of 75 adolescents.

#### 2.3.2. PA and Social Network Assessment

**Figure 2:**
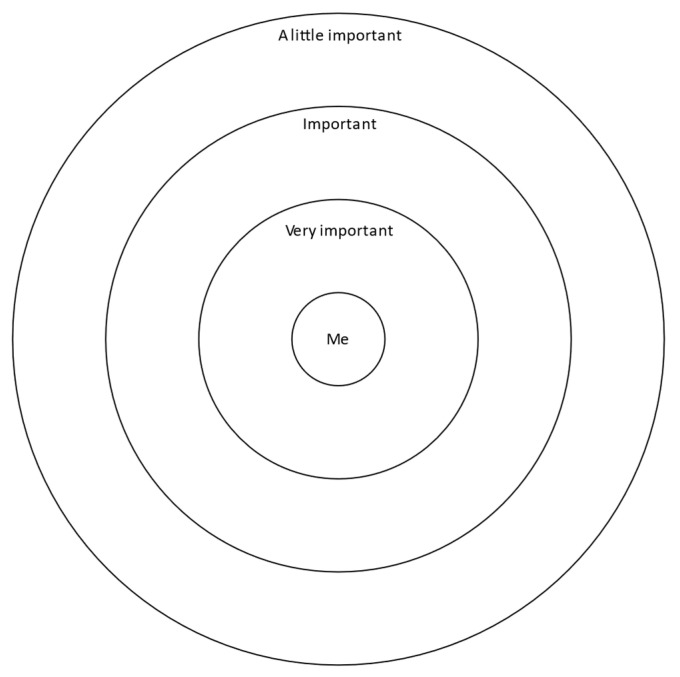
Example of three concentric circles facilitating the Convoy Model derived from Kahn and Antonucci [35].

**Figure 3:**
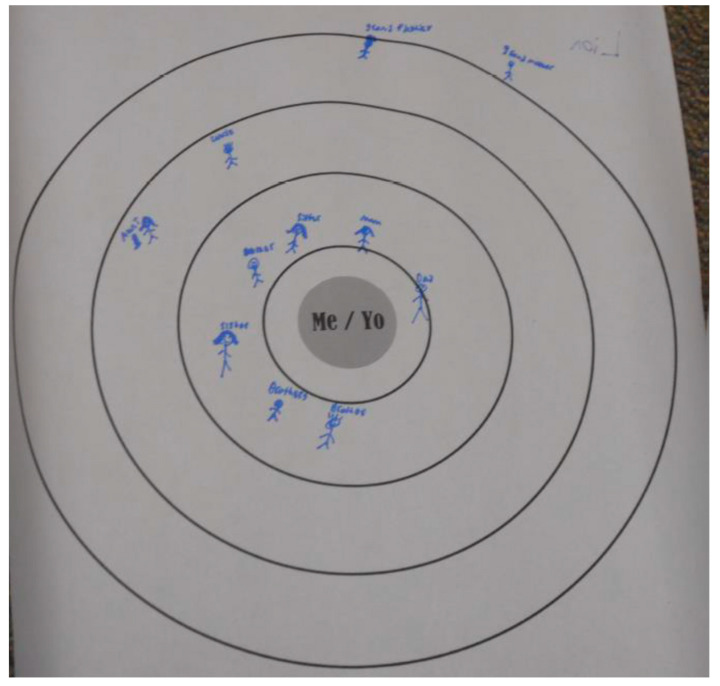
Example of Convoy Model filled out during a focus group discussion.

This study utilized a Convoy Model approach to foster focus group conversation among adolescents. The Convoy Model is a way of collecting egocentric network data and is based in theories of attachment. The method consists of drawing three concentric circles on a paper with the ego (adolescent) at the center [35,36]. See Figure 2 for an example of a Convoy Model diagram and Figure 3 for a completed example. This method allows for more tactile and visual responses to social networks, as well as serving as a good activity to foster conversation [37]. The adolescents were instructed to “draw people who are physically active or actively play with you most frequently. This could be PA, exercise, active games, walking, and/or sports. Put the people closest to you in the circle closest to you and those who you are active with less in the outer circles. The circle closest to you will be the people that often (5 days or more per week) spend time with you with PA or are most important to you in PA. The next circle will be those people that spend time with you, like 3 or 4 times per week, and the outer circle will be the people that hardly spend time with you during physical activities or are least important for you in PA.” Adolescents were also asked to draw a small star next to the people that are most important or most fun. After the adolescents were done drawing, *promotora*-researchers asked them to tell the group about the people in their circles. These data can be used to generate network composition measures, similar to a name generator and name interpreter approach; however, the stated aim of this activity was to foster conversation among the adolescents regarding social influence, healthy eating, and active living. Hence, the data generated were qualitative in nature and were analyzed as such using a phenomenological approach grounded in attachment and social support theories [38].

### 2.4. Case Study 4: ¡Haz Espacio para Papi! Health Program and Network Change

#### 2.4.1. Study Description and Sample

This case study was a part of the larger *Salud Para Usted y Su Familia* (SPUSF) [Health for You and Your Family] parent project which utilized a *promotora*-researcher model to develop, implement, and evaluate a father-focused, family-centered program based on active living, healthy eating, and family communication tailored to Mexican-heritage families living in *colonia* areas along the south Texas border with Mexico. *Promotora*-researchers (or *promotoras*) are members of the community trained in health promotion and research techniques to bridge the gap between academic researchers and the community [39]. The *Haz Espacio para Papi!* (Make Room for Daddy!; HEPP) program outcomes (e.g., nutrition and PA) focused on fathers and adolescents (9–11 years old); however, mothers were engaged in the program, as they are often considered family gatekeepers in this cultural context and tend to be responsible for adolescent caregiving [40]. A modified stepped-wedge cluster randomized trial design was used to maximize benefit to participating families and reduce the amount of resources needed to implement the program [41]. A research team consisting of *promotoras* and academic researchers worked together to create and refine the curriculum through an iterative process of creation, modification, and testing [42]. Adolescents were surveyed by *promotoras* before and after the health program.

#### 2.4.2. PA Assessment

ActiGraph GT9X accelerometers (ActiGraph Corporation, Pensacola, FL, USA) were used to measure daily time spent in sedentary, light, and MVPA. Adolescents were asked to wear the monitor on their non-dominant wrist 24 h per day for 7 days. Full details on accelerometer processing can be found elsewhere [43]. In short, non-wear periods were identified according to the procedures established by Ahmadi et al. [44]. To be included in the analysis, adolescents were required to have at least four valid monitoring days, with at least one of those days being a weekend day. Monitoring days were considered valid if wear time was greater than 960 min per day. A machine-learned random forest classifier that was specifically designed and validated for assessing PA in school-aged youths [45,46] was used to determine activity. In the current study, MVPA was defined as the sum of daily time spent walking, running, and engaging in moderate-to-vigorous activities and games. Minutes of MVPA and sedentary minutes were then averaged across all valid wear days to produce a mean MVPA per day and mean sedentary minutes per day for each adolescent.

#### 2.4.3. SNA Approach

As this study took an egocentric approach, adolescents reported up to five individuals with whom they had “actively played with most often” in the previous month. This list was limited to five names to capture the most salient connections while reducing respondent burden [24]. Adolescents were also asked name interpreters for each alter: the sex of each alter, their relationship to each alter (sibling, mother, father, friend, grandparent, aunt/uncle, cousin, other), what activities they did most often with each alter (open ended response), and frequency with which they played with each alter (“once in a while”, “sometimes”, “often”). In any instance that children did not state their mother and father in their network, specific questions were asked regarding their mother and father as this was a family focused program. An electronic copy of the English version of the social network collection tool can be found in Table S3.

## 3. Results

### 3.1. Case Study 1: Youth Engagement in Sport

At baseline, 74 adolescents provided some data; however, only 46 (62.2%) provided network data. At the 3-month timepoint, 34 adolescents provided data and 22 (64.7%) provided network data. At the 6-month timepoint, 32 adolescents provided data and 17 (53.1%) provided network data. Of those who provided network data, adolescents reported a mean of 1.98 (SD = 1.47) peers at baseline, 2.32 (SD = 1.70) at 3-months, and 2.53 (SD = 1.77) at 6-months. Adolescents also reported that their friends were active around 2 h per week at baseline (M = 2.42; SD = 1.65) and 3-months (M = 2.67; SD = 1.60) but roughly 3 h per week at 6-months (M = 3.28, SD = 1.47). As measured by the IPAQ-SF, adolescents reported a mean of 3365 MET min (SD = 3801.62) per week at baseline, 3942.45 MET min (SD = 5451.69) per week at 3-months, and 6519.24 MET min (SD = 7348.38) per week at 6-months.

### 3.2. Case Study 2: Summer Care Program Social Networks

For the egocentric portion of the study, a total of 182 adolescents (46.2% male) were surveyed at time 1 and 175 adolescents (46.8% male) at time 2. Participants were, on average, 9.93 years old (SD = 1.28) and reported being active an average of 2–3 h per week (M = 3.25; SD = 1.41) at time 1. Adolescents were, on average, 9.73 years old (SD = 1.30) and again reported being active 2–3 h per week, on average (M = 3.38; SD = 1.40). In time 1, adolescents reported a mean of 3.30 (SD = 1.50) connections in the SCP and 3.51 (SD = 1.63) outside of the program. Participants perceived relatively similar levels of PA in both in-program (M = 2.82, SD = 1.54) and outside (M = 2.80, SD = 1.50) networks. They also reported playing often with 1.71 (SD = 1.49) other adolescents in the program and 1.86 (SD = 1.57) outside the program. At time 2, participants reported a mean of 3.99 (SD = 1.60) connections in the SCP and 3.76 (SD = 1.45) outside of the program. They perceived relatively similar levels of PA in both in-program (M = 2.79, SD = 1.19) and outside (M = 2.99, SD = 1.38) networks. Adolescents also reported playing often with 2.29 (SD = 1.74) other adolescents in the program and 2.01 (SD = 1.60) outside the program. Further sample characteristics can be found in Table 2. 

For the whole network portion of the study, two separate networks were created based on SCP sites. At site one, 100 adolescents were surveyed at time one and 77 were surveyed at time two. At site two, 27 adolescents were surveyed at time one and 32 were surveyed at time two. Participants at site one nominated a mean of 2.92 (SD = 1.02) others at time one and 2.81 (SD = 1.25) at time two. Participants at site two nominated a mean of 2.31 (SD = 1.80) others at time one and 2.67 (SD = 1.40) at time two. Network density ranged from 0.03 and 0.07. Reciprocity ranged from 0.31 and 0.45. Transitivity ranged from 0.22 and 0.32. 

### 3.3. Case Study 3: Convoy Method among Colonia Youth: Qualitative

Many of the adolescents decided to group people in their social network by specific activity or by the location of that activity. For example, they would talk about the people with whom they would play soccer or tag or walk to the park, and cluster each around that activity. Many adolescents talked about co-participation in sports activities as well as active play or active transportation activities, indicating tangible conditional social support. While the discussion was specific to physical activities, many adolescents talked about sedentary activities such as playing video games or watching TV with others. In these discussions, several of the adolescents mentioned specific people that were more sedentary than others, indicating that certain individuals in their network were more influential in their sedentary habits as opposed to their PA behaviors. 

Siblings or friends who lived nearby were mentioned frequently, indicating proximal social influence; however, the participants listed many extended family members as having influence and importance in PA. Parents were mentioned as providers of several forms of support, such as tangible instrumental support by transportation and intangible motivational support. Coaches or teachers were also mentioned but were more often listed toward the outside of the circles. It is important to also mention that in some situations, children would put a star next to a family member that was not placed in the inner circles, signifying these were very important people to them that they did not get to spend much time with. This was valuable information, since some of the children reflected on this during the discussion, realizing this was a wake-up call for them and those family members to be more effortful in their time spent together.

When asked why they placed certain individuals in different circles, many adolescents described elements of time spent together, fun or enjoyment with the person, and tangible help or support for being active. These results indicate that participants in the discussions judged several concepts or constructs of social influence when considering who to list in their personal social networks. 

### 3.4. Case Study 4: ¡Haz Espacio para Papi! Health Program and Network Change

Overall, 42 adolescents (M age = 9.79, SD = 1.01; 54.8% girls) completed both pre- and post-intervention evaluations. Before the program, participants reported 174 total alters: 70.3% of the alters reported were of the same sex as the adolescent displaying gender homophily. Friends (37.1% of alters) and siblings (24.6%) were reported most often, with similar frequencies being reported after the program. Adolescents in this sample attained a mean of 50.90 min of MVPA per day (SD = 16.20) before the intervention and 56.01 min (SD = 18.96) after the intervention. Adolescents reported significantly more alters they had actively played with before the program (M = 4.38; SD = 1.00) as compared to after the program (M = 3.83; SD = 1.32; t(41) = 2.60, *p* = 0.01). However, there was no significant difference in the average frequency with which the adolescents reported playing with their alters between before (M = 1.18; SD = 0.65) and after the program (M = 1.29; SD = 0.67); t(41) = −0.98, *p* = 0.33. In this context, adolescents reported a similar average frequency of play across their networks; however, multilevel models are needed to better detect dyadic differences in frequency of play over time. 

## 4. Discussion

This paper aimed to describe several ways in which researchers could collect SNA and PA data regarding minority adolescents. Through these case studies, we hope to exemplify the variety and utility of SNA designs to understand PA among adolescents and encourage its use among PA researchers. Case studies presented here provide examples of ways to evaluate social networks via whole network and/or egocentric network research approaches, and how social connections might associate with PA among adolescents. Descriptive statistics were provided for each case study; however, more advanced analysis techniques can be used to answer robust research questions. Examples of these analyses are provided below. 

To further analyze egocentric data in Case Studies 1, 2, and 4, analysis plans could include alter-level or ego-level analysis. Alter-level analysis is conducted when the outcome of interest (i.e., dependent variable) is reported for each specific alter in the network [25]. For example, an adolescent might indicate five people they feel close to, and how often they are active with or play with each of their five alters. In this case, a researcher could analyze factors (e.g., alter’s age; alter’s relation to ego; composition of ego’s network that is active) related to an adolescent reporting higher levels of active play with an alter [47]. In this case, each alter in the network would have a data point which would be clustered within each ego, necessitating the use of multilevel models [25,47]. Conversely, alter-level data can be aggregated to the ego-level by creating network composition scores, such as average level of PA among network members or percent of network members who provide support [25]. Aggregating the data in this way loses granularity between unique alters but provides the opportunity for to assess ego-level outcomes, such as ego’s PA levels [25]. For example, a researcher could examine what network properties (e.g., average PA score across all alters; proportion of the network that are kin) are associated with higher PA for adolescents. Subsequently, regression analyses could be used to determine significant associations between ego PA and network composition scores while controlling for other variables [43,48]. Differences in composition scores can also be created to determine change in networks over time [49]. 

To further analyze whole network data in Case Study 2, analysis plans could include several whole network modeling procedures. Exponential Random Graph Models help determine factors associated with the presence of friendship connections between adolescents in the networks at each independent time point [50]. Specifically, this could detail whether PA was associated with tie presence [51]. Separable Temporal Exponential Random Graph Model can determine significant factors in formation or maintenance of ties over time [52]. This modeling procedure could determine if adolescents with higher PA are more likely to form and/or maintain connections over time [51]. Linear Network Autocorrelation Models determine significant associations between individual PA and the PA of one’s connections in the network controlling for other factors [53]. This model would be able to determine if adolescents’ PA or skill competency was associated with that of their direct connections’ [54,55,56]. Stochastic Actor-Oriented Models determine the probabilities associated with the actor making particular changes (or no change) to their social ties or behavior based on the empirical data presented and parameters selected by the researcher [57,58]. Coevolution of behavior and network models would enable researchers to analyze adolescents’ influence on peers’ PA while considering the complexity of interdependent network data. These models have been used in prior studies to understand PA in longitudinal data [9,59] and have the capability to enhance our understanding of diffusion effects by modeling social selection, social influence, and behavior change over time.

In Case Study 3, we examined the convoy method for engaging adolescents in a conversation about their networks. While not explicitly demonstrated here, the convoy method can also be used in quantitative data collection and as an intervention activity. The convoy method can be used as a network generator approach to elicit a list of network members while also denoting levels (ordinal measurement) of closeness or another dimension of choice [36]. These drawings can then be scanned and turned into network composition measurements. Data collection software such as Network Canvas have taken this method to provide a more tactile approach to electronically collecting network data [60,61]. Additionally, utilizing the convoy method to foster discussion around social influence could be used in intervention programs seeking to leverage and/or impact the social structure surrounding the adolescents [36]. In this way facilitators could ask directed reflection questions about how the adolescents might use members in their network to be more active, how they might be able to influence others, and how they think others influence themselves. 

Although Case Studies 1 and 4 utilized SNA within an intervention design for evaluation purposes, PA interventions have rarely employed specific social network approaches in their designs [8]. Valente [62] hypothesized four effective approaches to leverage networks for health interventions: (1) opinion leaders—engaging individuals who are selected on the basis of a network property who may have more influence throughout the network such as providing information or support or through popularity; (2) segmentation—engaging certain groups of people to reinforce desired behaviors within the group; (3) induction—encouraging peer-to-peer interactions to promote diffusion of effects to other network members; and (4) alteration—changing the network by adding or deleting members, adding or deleting specific social ties, or changing the entire network. Past PA research has used the opinion leaders approach by selecting peer influencers to promote PA [63,64]. In a pair of studies by van Woudenberg [64,65], researchers trained opinion leaders, selected based on closeness centrality score, to promote PA first in person and then through vlogs or video blogs. These approaches alone did not significantly improve the average level of PA throughout the network as hypothesized in agent-based models [64,65]. The further use of other network approaches is needed in the design of interventions as well as the evaluation of intervention effects.

### 4.1. Limitations

It should be noted that the case studies provided here are not the only ways SNA can be used in PA research; however, they represent a variety of common ways the techniques can be applied. Likewise, the analysis suggestions in the discussion section are not an exhaustive list but provide the reader with options and ideas for future reading and research. 

### 4.2. Implications

The role of social influence in adolescent PA research is not new and has been hypothesized in many different models and theories. Likewise, the measurement of social influence can incorporate many constructs and latent variables. However, the true implication of this work is to promote and advocate for defining and measuring specific social connections as they relate to adolescent PA. Increasingly, research calls for granular, detailed approaches to measurement and contextually specific approaches to interventions. By using SNA, researchers can measure granular, detailed data on specific social relationships which make up an individual’s social context. Hence, by understanding the social context in a more specific manner, researchers will be more poised to intervene on such influences. Further, by providing case studies here as a primer for future learning, we hope to promote the use of SNA in more research, especially in the design and evaluation of interventions. 

## 5. Conclusions

Numerous theories involve the concept of social influence and consider how the social environment may impact health behavior. SNA is a powerful tool with which to investigate these constructs. This article not only provides tangible ways to collect SNA data but also potential analyses which could be used to evaluate social influences on PA behaviors. By understanding how SNA data can be collected in relation to PA, this article aims to encourage more refined use of this technique in PA research, including future intervention programs using network approaches.

## Figures and Tables

**Table 1:** Summary of network measures and terms.

Network Measure/Term	Definition
Ego	Denotes the person being surveyed in person-centered (egocentric) network studies
Alter	People within a person’s network
**Centrality**	A set of measures calculated on each node in a network indicating the levels of connection and potential power, influence, and popularity that a given node has relative to others in the network
Degree	A specific measure of centrality which counts the number of links to and from a node in a network; nodes with higher degree scores have more connections within their network and, therefore, may be more popular, powerful, or influential.
Closeness	A specific measure of centrality that reveals the average distance between the nodes in a network. Nodes with high closeness scores are more reachable and may be more depended on in the network
Betweenness	A specific measure of centrality indicating the frequency to which a node lies on the shortest path connecting everyone else within the network. Nodes with high betweenness often serve as important connection points between others in the network, and therefore, may have a lot of control over the diffusion of ideas or behaviors
**Groups**	A set of at least three people who are more closely connected to each other than other people in a larger network
Components	A set of nodes that are linked to one another through paths of any length
K-Cores	A maximal subgraph (inclusion of all nodes and ties that meet a certain criteria) in which each point is adjacent to *k* other points. For example, a 4k-core would reveal a subgraph of nodes that all have degree scores of 4 or higher
Modularity/Community Detection	Measure of how well groups characterize a network, i.e., how well do nodes fit into non-overlapping groups
**Network Structure**	A measure of a network in its entirety; this describes the structure of the overall network
Size	A count of all nodes in the network
Density	Number of connections in the network reported as a fraction of the total possible links Higher density scores reveal a more densely connected network
Centralization	Degree a network’s ties, focused on one person or set of people Higher centralization scores reveal a more hierarchically structured network
**Homophily**	The tendency for two people to connect based on a shared characteristic or trait: “birds of a feather flock together”
Social Selection	The “cause” of a homophilous tie, where someone chooses to connect with another person because of a common trait/characteristic
Social Influence	The “effect” of a social tie resulting in homophily, where someone becomes more like the person they are connected to over time and comes to share common traits/characteristics
**Network Composition**	Proportion of an egocentric network that holds a certain characteristic, belief, or attribute, or engages in a particular behavior (e.g., the proportion of a network that identifies as female)
Network Exposure	A specific measure of composition to determine the proportion of an individual’s network that meets a certain criterion, therefore “exposing” the ego to that criterion (e.g., the proportion of someone’s network that exercises 5 days per week)

*Note.* All definitions were adapted from Valente’s (2010) *Social Networks and Health.*

**Table 2:** Case study designs and sample characteristics.

	(1) Youth Engagement in Sport	(2) Summer Care Program	(3) Convoy Model	(4) Family Focused Intervention
Description	Students from three inner city, low-income, minority serving middle schools (aged 10–13) in Kansas City, Missouri were recruited to participate in an after-school, sports sampling intervention with the main goal of increasing physical activity.	Adolescents aged 8–12 years old from two summer care programs (i.e., Boys & Girls Clubs) were invited to participate in researcher administered surveys at the start (time 1) and end (time 2) of summer (8 weeks between time points).	This study utilized a Convoy Model approach to foster focus group conversation among adolescents aged 7–11 years old from *colonias* on the border of Texas and Mexico.	This project utilized a *promotora*-researcher model to develop, implement, and evaluate a father-focused, family-centered program based on active living, healthy eating, and family communication available to Mexican-heritage families living in *colonia* areas along the south Texas border with Mexico.
Study Design	Intervention	Longitudinal	Cross-Sectional	Intervention
Paradigm	Quantitative	Quantitative	Qualitative	Mixed-Methods
Network Approach	Egocentric	Whole network and Egocentric	Egocentric	Egocentric
Network Generator	Please list the first and last name for up to 5 of the friends whom you feel closest to (spend your time with) at your school.	For the next few items please think about the people you hang around with, talk to, and do things with the most here at the Boys and Girls Club. When I ask about “active play” I mean activities that involve moving or that makes you breathe harder or makes your heartbeat faster. Please use the roster and tell me the names of up to five people you hang around with, talk to, and do things with the most here.	Draw people who are physically active or actively play with you and who are important to you all for physical activity, exercise, active games, walking, and/or sports. Put the people closest to you in the circle closest to you and those who are less important in the outer circles. The circle closest to you will be the people that often (5 days or more per week) spend time with you with physical activity or are most important to you in physical activity. The next circle will be those people that spend time with you, like 3 or 4 times per week, and the outer circle will be the people that hardly spend time with you during physical activities or are least important for you in physical activity.	For the next few items please think about the people you are physically active with and actively played with most often in the last month. You do not have to give me the person’s actual name as long as you can remember who you are talking about when answering questions. Please tell me the names of up to five people you are physically active with and actively played with most often in the last month.
PA Measure	Self-report	Self-report	Self-report	Accelerometer
Sample Size	74	182	75	42
Age	-	9.93 years old (SD = 1.28)	9.97 years old (SD = 1.42)	9.79 years old (SD = 1.01)
Sex				
Boy	51.4%	46.2%	49.3%	45.2%
Girl	48.6%	53.8%	50.7%	54.8%
Race/Ethnicity				
African American/Black	51.4%	48.4%	-	-
White, non-Hispanic	21.5%	14.6%	-	-
Hispanic/Latinx	17.6%	33.7%	-	-
Some other race	9.5%	3.3%	-	-

*Note.* Race and ethnicity information not available for case studies 3 and 4; however, adolescents needed to be of Mexican heritage (grandparent, parent, and/or adolescent born in Mexico) to participate. Similarly, age was not recorded for case study 1.

## Data Availability

The data presented in this study are available on request from the corresponding author.

## Supplementary Materials

The following supporting information can be downloaded at: https://www.mdpi.com/article/10.3390/ijerph191811545/s1, Table S1: Youth Engagement in Sport Survey; Table S2: Summer Care Survey; Table S3: Health for you and your Family Survey.
