# Supplementary material for: Conducting Physical Activity Research on Racially and Ethnically Diverse Adolescents Using Social Network Analysis: Case Studies for Practical Use
Source: Int J Environ Res Public Health. 2022 Sep 14;19(18):11545. doi: 10.3390/ijerph191811545 (PMC9517360; doi:10.3390/ijerph191811545)
Supplement: Supplementary file 1 [file ijerph-19-11545-s001.zip › ijerph-1905226-supplementary.pdf]

Table S1: Youth Engagement in Sport Survey

Social Network

Next, we will be asking you questions regarding your friends. We would love to be able to put you in groups with your friends, but this may not always be possible. Please list the first and last name for up to 5 of the friends whom you feel closest to (spend your time with) at your school. We would like to ask you some further questions about these people.

	**Person 1**	**Person 2**	**Person 3**	**Person 4**	**Person 5**
**a.** Person X Initials / Name					
**b.** Out of these friends, who's opinion do you respect the most in the group?	☐	☐	☐	☐	☐
**c.** How many hours per week do you think this friend is physically active?	☐ None<br>☐ About half an hour<br>☐ About one hour<br>☐ About 2-3 hours per week<br>☐ About 4-6 hours<br>☐ About 7 hours or more	☐ None<br>☐ About half an hour<br>☐ About one hour<br>☐ About 2-3 hours per week<br>☐ About 4-6 hours<br>☐ About 7 hours or more	☐ None<br>☐ About half an hour<br>☐ About one hour<br>☐ About 2-3 hours per week<br>☐ About 4-6 hours<br>☐ About 7 hours or more	☐ None<br>☐ About half an hour<br>☐ About one hour<br>☐ About 2-3 hours per week<br>☐ About 4-6 hours<br>☐ About 7 hours or more	☐ None<br>☐ About half an hour<br>☐ About one hour<br>☐ About 2-3 hours per week<br>☐ About 4-6 hours<br>☐ About 7 hours or more
**d.** How many hours per week do you think this friend is physically active?	☐ None<br>☐ About half an hour<br>☐ About one hour<br>☐ About 2-3 hours per week<br>☐ About 4-6 hours<br>☐ About 7 hours or more	☐ None<br>☐ About half an hour<br>☐ About one hour<br>☐ About 2-3 hours per week<br>☐ About 4-6 hours<br>☐ About 7 hours or more	☐ None<br>☐ About half an hour<br>☐ About one hour<br>☐ About 2-3 hours per week<br>☐ About 4-6 hours<br>☐ About 7 hours or more	☐ None<br>☐ About half an hour<br>☐ About one hour<br>☐ About 2-3 hours per week<br>☐ About 4-6 hours<br>☐ About 7 hours or more	☐ None<br>☐ About half an hour<br>☐ About one hour<br>☐ About 2-3 hours per week<br>☐ About 4-6 hours<br>☐ About 7 hours or more
**e.** How many hours per week do you think this friend is physically active?	☐ None<br>☐ About half an hour<br>☐ About one hour<br>☐ About 2-3 hours per week<br>☐ About 4-6 hours<br>☐ About 7 hours or more	☐ None<br>☐ About half an hour<br>☐ About one hour<br>☐ About 2-3 hours per week<br>☐ About 4-6 hours<br>☐ About 7 hours or more	☐ None<br>☐ About half an hour<br>☐ About one hour<br>☐ About 2-3 hours per week<br>☐ About 4-6 hours<br>☐ About 7 hours or more	☐ None<br>☐ About half an hour<br>☐ About one hour<br>☐ About 2-3 hours per week<br>☐ About 4-6 hours<br>☐ About 7 hours or more	☐ None<br>☐ About half an hour<br>☐ About one hour<br>☐ About 2-3 hours per week<br>☐ About 4-6 hours<br>☐ About 7 hours or more
**f.** Does this friend encourage you to be physically active (e.g. suggest going to the park, playing	☐ Often<br>☐ Sometimes<br>☐ Never	☐ Often<br>☐ Sometimes<br>☐ Never	☐ Often<br>☐ Sometimes<br>☐ Never	☐ Often<br>☐ Sometimes<br>☐ Never	☐ Often<br>☐ Sometimes<br>☐ Never

basketball or soccer)?					
**g.** How close do you feel with each of these friends?	☐ Very close<br>☐ Somewhat close<br>☐ Close<br>☐ Not very close<br>☐ Not at all close	☐ Very close<br>☐ Somewhat close<br>☐ Close<br>☐ Not very close<br>☐ Not at all close	☐ Very close<br>☐ Somewhat close<br>☐ Close<br>☐ Not very close<br>☐ Not at all close	☐ Very close<br>☐ Somewhat close<br>☐ Close<br>☐ Not very close<br>☐ Not at all close	☐ Very close<br>☐ Somewhat close<br>☐ Close<br>☐ Not very close<br>☐ Not at all close

Table S2: Summer Care Survey

For the next few items please think about the people you **hang around with, talk to, and do things with the most here at the Boys and Girls Club**. When I ask about “active play” I mean activities that involve moving or that makes you breathe harder or makes your heartbeat faster. Please use the roster and tell me the names of **up to five people** you hang around with, talk to, and do things with the most here. (There is no minimum requirement. If participant is done nominating people leave questions blank)

**18-22.**	**18. Person 1**	**19. Person 2**	**20. Person 3**	**21. Person 4**	**22. Person 5**
**a.** Person X Initials / Name					
**b.** Is [Person X] a boy or girl?	☐ Boy<br>☐ Girl	☐ Boy<br>☐ Girl	☐ Boy<br>☐ Girl	☐ Boy<br>☐ Girl	☐ Boy<br>☐ Girl
**c.** What is your relationship to [Person X]? (Are they your…)	☐ Sibling<br>☐ Friend<br>☐ Relative<br>Other: _____	☐ Sibling<br>☐ Friend<br>☐ Relative<br>Other: ____	☐ Sibling<br>☐ Friend<br>☐ Relative<br>Other: ____	☐ Sibling<br>☐ Friend<br>☐ Relative<br>Other: ____	☐ Sibling<br>☐ Friend<br>☐ Relative<br>Other: ____
**d.** How old is [Person X]?					
**e.** Does [Person X] live….?	☐ In your household<br>☐ In your neighborhood<br>☐ Outside your neighborhood<br>I don’t know	☐ In your household<br>☐ In your neighborhood<br>☐ Outside your neighborhood<br>I don’t know	☐ In your household<br>☐ In your neighborhood<br>☐ Outside your neighborhood<br>I don’t know	☐ In your household<br>☐ In your neighborhood<br>☐ Outside your neighborhood<br>I don’t know	☐ In your household<br>☐ In your neighborhood<br>☐ Outside your neighborhood<br>I don’t know
**f.** What activities do you do with [Person X] most often?					
**g.** How often do you actively play with [Person X]?	☐ Often<br>☐ Sometimes<br>☐ Never	☐ Often<br>☐ Sometimes<br>☐ Never	☐ Often<br>☐ Sometimes<br>☐ Never	☐ Often<br>☐ Sometimes<br>☐ Never	☐ Often<br>☐ Sometimes<br>☐ Never
**h.** How many hours per week do you think [Person X] usually exercises in their free time, so much that they get out of breath or sweat?	☐ None<br>☐ About half an hour<br>☐ About one hour<br>☐ About 2-3 hours per week<br>☐ About 4-6 hours	☐ None<br>☐ About half an hour<br>☐ About one hour<br>☐ About 2-3 hours per week<br>☐ About 4-6 hours	☐ None<br>☐ About half an hour<br>☐ About one hour<br>☐ About 2-3 hours per week<br>☐ About 4-6 hours	☐ None<br>☐ About half an hour<br>☐ About one hour<br>☐ About 2-3 hours per week<br>☐ About 4-6 hours	☐ None<br>☐ About half an hour<br>☐ About one hour<br>☐ About 2-3 hours per week<br>☐ About 4-6 hours

	☐ About 7 hours or more<br>☐ I don't know	☐ About 7 hours or more<br>☐ I don't know	☐ About 7 hours or more<br>☐ I don't know	☐ About 7 hours or more<br>☐ I don't know	☐ About 7 hours or more<br>☐ I don't know
**i.** [Person X] helps me to be physically active?	☐ Yes<br>No	☐ Yes<br>☐ No	☐ Yes<br>☐ No	☐ Yes<br>☐ No	☐ Yes<br>☐ No
**j.** Does [Person X] know…		☐ Person 1	☐ Person 1<br>☐ Person 2	☐ Person 1<br>☐ Person 2<br>☐ Person 3	☐ Person 1<br>☐ Person 2<br>☐ Person 3<br>☐ Person 4

For the next few items please think about the people you spend the most time with outside of the Boys & Girls Club. Please tell me the names or initials of **up to five people** you spend the most time with. You do not have to give me the person's actual name as long as you can remember who you are talking about when answering questions. You are free to use that person's initials or a nickname if you want.

**23-27.**	**23. Person 1**	**24. Person 2**	**25. Person 3**	**26. Person 4**	**27. Person 5**
**a.** Person X Initials / Name					
**b.** Is [Person X] a boy or girl?	☐ Boy<br>☐ Girl	☐ Boy<br>☐ Girl	☐ Boy<br>☐ Girl	☐ Boy<br>☐ Girl	☐ Boy<br>☐ Girl
**c.** What is your relationship to [Person X]? (Are they your…)	☐ Mother<br>☐ Father<br>☐ Sibling<br>☐ Friend<br>☐ Relative<br>☐ Other: ______	☐ Mother<br>☐ Father<br>☐ Sibling<br>☐ Friend<br>☐ Relative<br>☐ Other: _____	☐ Mother<br>☐ Father<br>☐ Sibling<br>☐ Friend<br>☐ Relative<br>☐ Other: _____	☐ Mother<br>☐ Father<br>☐ Sibling<br>☐ Friend<br>☐ Relative<br>☐ Other: _____	☐ Mother<br>☐ Father<br>☐ Sibling<br>☐ Friend<br>☐ Relative<br>☐ Other: _____
**d.** How old is [Person X]?					
**e.** Does [Person X] live….?	☐ In your household<br>☐ In your neighborhood<br>☐ Outside your neighborhood<br>☐ I don't know	☐ In your household<br>☐ In your neighborhood<br>☐ Outside your neighborhood<br>☐ I don't know	☐ In your household<br>☐ In your neighborhood<br>☐ Outside your neighborhood<br>☐ I don't know	☐ In your household<br>☐ In your neighborhood<br>☐ Outside your neighborhood<br>☐ I don't know	☐ In your household<br>☐ In your neighborhood<br>☐ Outside your neighborhood<br>☐ I don't know
**f.** What activities do you do with [Person X] most often?					
**g.** How often do you actively play with [Person X]?	☐ Often<br>☐ Sometimes<br>☐ Never	☐ Often<br>☐ Sometimes<br>☐ Never	☐ Often<br>☐ Sometimes<br>☐ Never	☐ Often<br>☐ Sometimes<br>☐ Never	☐ Often<br>☐ Sometimes<br>☐ Never

**h.** How many hours per week do you think [Person X] usually exercises in their free time, so much that they get out of breath or sweat?	☐ None<br>☐ About half an hour<br>☐ About one hour<br>☐ About 2-3 hours per week<br>☐ About 4-6 hours<br>☐ About 7 hours or more<br>☐ I don't know	☐ None<br>☐ About half an hour<br>☐ About one hour<br>☐ About 2-3 hours per week<br>☐ About 4-6 hours<br>☐ About 7 hours or more<br>☐ I don't know	☐ None<br>☐ About half an hour<br>☐ About one hour<br>☐ About 2-3 hours per week<br>☐ About 4-6 hours<br>☐ About 7 hours or more<br>☐ I don't know	☐ None<br>☐ About half an hour<br>☐ About one hour<br>☐ About 2-3 hours per week<br>☐ About 4-6 hours<br>☐ About 7 hours or more<br>☐ I don't know	☐ None<br>☐ About half an hour<br>☐ About one hour<br>☐ About 2-3 hours per week<br>☐ About 4-6 hours<br>☐ About 7 hours or more<br>☐ I don't know
**i.** [Person X] helps me to be physically active?	☐ Yes<br>☐ No	☐ Yes<br>☐ No	☐ Yes<br>☐ No	☐ Yes<br>☐ No	☐ Yes<br>☐ No
**j.** Does [Person X] know…		☐ Person 1	☐ Person 1<br>☐ Person 2	☐ Person 1<br>☐ Person 2<br>☐ Person 3	☐ Person 1<br>☐ Person 2<br>☐ Person 3<br>☐ Person 4

Table S3: Health for you and your Family Survey

For the next few items please think about the people you are **physically active with and actively played with most often in the last month**. You do not have to give me the person's actual name as long as you can remember who you are talking about when answering questions. Please tell me the names of **up to five people** you are physically active with and actively played with most often in the last month.

**30-34.**	**30. Person 1**	**31. Person 2**	**32. Person 3**	**33. Person 4**	**34. Person 5**
**a.** Person X Initials / Name					
**b.** Is [Person X] a boy or girl?	☐ Boy<br>☐ Girl	☐ Boy<br>☐ Girl	☐ Boy<br>☐ Girl	☐ Boy<br>☐ Girl	☐ Boy<br>☐ Girl
**c.** What is your relationship to [Person X]?	☐ Mother<br>☐ Father<br>☐ Sibling<br>☐ Friend<br>☐ Grandparent<br>☐ Aunt / Uncle<br>☐ Cousin<br>☐ Other	☐ Mother<br>☐ Father<br>☐ Sibling<br>☐ Friend<br>☐ Grandparent<br>☐ Aunt / Uncle<br>☐ Cousin<br>☐ Other	☐ Mother<br>☐ Father<br>☐ Sibling<br>☐ Friend<br>☐ Grandparent<br>☐ Aunt / Uncle<br>☐ Cousin<br>☐ Other	☐ Mother<br>☐ Father<br>☐ Sibling<br>☐ Friend<br>☐ Grandparent<br>☐ Aunt / Uncle<br>☐ Cousin<br>☐ Other	☐ Mother<br>☐ Father<br>☐ Sibling<br>☐ Friend<br>☐ Grandparent<br>☐ Aunt / Uncle<br>☐ Cousin<br>☐ Other
If other please list here.					
**d.** What activities do you do with this person most often?					
**e.** How often do you actively play with [Person X]?	☐ Often<br>☐ Sometimes<br>☐ Never	☐ Often<br>☐ Sometimes<br>☐ Never	☐ Often<br>☐ Sometimes<br>☐ Never	☐ Often<br>☐ Sometimes<br>☐ Never	☐ Often<br>☐ Sometimes<br>☐ Never
**f.** [Person X] is physically active regularly?	☐ Yes<br>☐ No<br>☐ I don't know	☐ Yes<br>☐ No<br>☐ I don't know	☐ Yes<br>☐ No<br>☐ I don't know	☐ Yes<br>☐ No<br>☐ I don't know	☐ Yes<br>☐ No<br>☐ I don't know
**g.** [Person X] helps me to be physically active?	☐ Yes<br>☐ No	☐ Yes<br>☐ No	☐ Yes<br>☐ No	☐ Yes<br>☐ No	☐ Yes<br>☐ No

If Mother or Father, or both were not mentioned in 29-34, Please ask these questions for missing person(s). If both were mentioned please skip to question 37.

	**35. Mother**	**36. Father**
**a.** What activities do you do with this person most often?		
**b.** How often do you actively play with [Person X]?	☐ Often<br>☐ Sometimes<br>☐ Never	☐ Often<br>☐ Sometimes<br>☐ Never
**c.** [Person X] is physically active regularly?	☐ Yes<br>☐ No<br>☐ I don't know	☐ Yes<br>☐ No<br>☐ I don't know
**d.** [Person X] helps me to be physically active?	☐ Yes<br>☐ No	☐ Yes<br>☐ No

If child responded YES that mother or father helped them to be active please ask these follow up questions. If not please skip to question 39.

**37.**	**If YES to mother,** how does your mother help you to be physically active?	
**38.**	**If YES to father,** how does your father help you to be physically active?	
